# Extrachromosomal Circular DNA MIRECD Enhances Necroptosis and Predicts Prognosis of Myocardial Infarction

**DOI:** 10.34133/research.0803

**Published:** 2025-08-08

**Authors:** Yiheng Zhao, Yujia Zhou, Shuchen Zhang, Boyang Xiang, Xiang Zhou

**Affiliations:** ^1^ Department of Cardiology, The Second Affiliated Hospital of Nanjing Medical University, Nanjing, China.; ^2^ Department of Cardiology, The Second Affiliated Hospital of Soochow University, Suzhou, China.

## Abstract

Recent researches have revealed the potential utility of extrachromosomal circular DNAs (eccDNAs) as biomarkers in various diseases. However, the association between plasma eccDNAs and myocardial infarction (MI) remains unclear. In this study, we extracted plasma eccDNA from blood samples of individuals with acute MI and conducted Circle-Seq. We identified an MI-related eccDNA (MIRECD) with high expression levels in the plasma of patients with MI. Sanger sequencing validated its loop construction and sequence. Mechanistically, MIRECD could aggravate necroptosis via regulating the expression of mixed-lineage-kinase-domain-like pseudokinase (MLKL). Kaplan–Meier analysis demonstrated that the incidences of major adverse cardiac events (MACEs) and cardiovascular mortality were higher in individuals with elevated MIRECD levels. Univariate and multivariate Cox regression analyses indicated that MIRECD independently predicted the occurrence of MACEs in patients with MI. The addition of MIRECD enhanced the discrimination and reclassification ability compared with conventional risk factors. In conclusion, our study identified a novel eccDNA, MIRECD, which might regulate myocardial necroptosis through MLKL. MIRECD has the potential to serve as a reliable indicator for predicting the prognosis and stratifying the risk of MI.

## Introduction

Myocardial infarction (MI), characterized by ischemic necrosis of cardiac tissue, is the primary cause of mortality globally [1]. Heart failure (HF) and arrhythmias are frequent complications of MI, considerably deteriorating the quality of many patient’s life and imposing substantial economic burdens on both society and individuals [2]. Therefore, it is of vital importance to discover appropriate markers to assess prognosis and provide new targets to improve myocardial tissue repair. Although cardiac troponin (cTn) and B-type natriuretic peptide (BNP) are established biomarkers recommended in clinical guidelines [3–5], several new biomarkers have been identified to facilitate the risk stratification for patients with MI [6,7].

Extrachromosomal circular DNAs (eccDNAs) is type of DNA that originates from chromosomes but which is free of chromosomal DNA once generated [8]. The formation of eccDNAs can be elucidated through 3 theoretical frameworks: chromothripsis, the breakage–fusion–bridge cycle, and the episome model [9]. eccDNAs are often shorter than 500 base pairs (bp) but can replicate and encode amino acids [10]. Both normal and cancer cells are reported to express eccDNAs, the function of which include amplification of genetic heterogeneity in tumors, contribution to drug resistance during treatment, expression of regulatory RNAs, and gene dose compensation [11,12]. Recent studies indicated that large eccDNAs (>1 kb) can be directly transcribed in nondividing cardiomyocytes, highlighting their potential involvement in gene expression. Compared to linear DNA with the same sequence, small circular DNA (<1 kb) elicited a more potent cytokine response. In addition, macrophages exhibited a more pronounced response compared to cardiomyocytes [13]. However, the precise molecular function of eccDNAs remains largely unexplored.

Considering the potential function of eccDNAs, several studies have proposed that eccDNAs could be used as biomarkers for disease diagnosis and prognosis. For example, Cai et al. [14] showed that circulating tumor DNA could reflect tumor heterogeneity and monitor therapeutic responses in patients with hepatocellular carcinoma. Cen et al. [15] confirmed significant down-regulation of the eccDNA DNMT1^circle10302690–10302961^ in high-grade serous ovarian cancer and showed that its expression was negatively related to prognosis. In addition to tumors, Lv et al. [16] detected the presence of eccDNA in urine samples from patients with advanced chronic kidney disease and revealed significantly higher urinary cell-free eccDNAs in patients with chronic kidney disease compared with healthy controls. Zhang et al. [17] found that serum eccDNA-chr2:131208878–131424362 levels were strongly elevated in patients with pulmonary arterial hypertension, suggesting that eccDNA may serve as a noninvasive biomarker for diagnosing this condition. However, it remains unclear whether eccDNAs are associated with other cardiovascular diseases.

This study utilized Circle-Seq on blood samples from patients with MI and healthy controls to identify differentially expressed eccDNAs. We ultimately identified a specific type of eccDNA, named MI-related eccDNA (MIRECD), and explored its potential mechanism. Furthermore, we conducted a cohort study to investigate the prognostic value of plasma eccDNA in patients with MI.

## Results

### The landscape of eccDNA in MI

To identify eccDNAs related to MI on genome-wide scale, we performed Circle-Seq on 10 blood samples from 5 patients with MI and 5 healthy controls. The baseline characteristics of the participants are shown in Table S1.

**Fig. 1.**
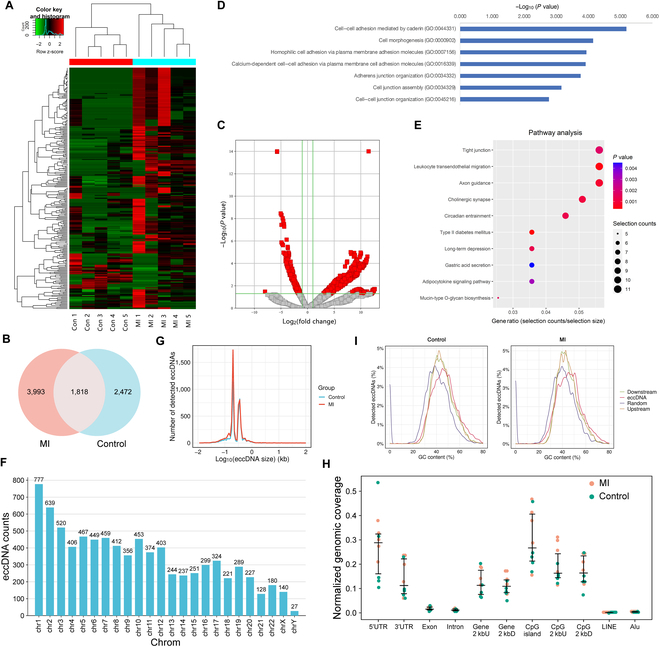
Identification of differentially expressed eccDNAs in plasma. (A) Hierarchical clustering showing the differentially expressed plasma eccDNAs in patients with MI. (B) Venn diagram showing the overlap of eccDNAs between MI and healthy controls. (C) Volcano plot showing differential abundant of eccDNAs between MI and control groups. GO enrichment analyses (D) and KEGG pathway analysis (E) were used to predict the biological functions and annotate functional pathways of plasma eccDNAs in patients with MI. (F) The distribution of eccDNAs in different chromosomes. (G) The length density distribution curve of eccDNAs. (H) Box plots showing the distribution of eccDNAs in different genomic regions. UTR, untranslated region. (I) GC content of the eccDNA locus and its adjacent regions, compared to the genomic average.

To elucidate the properties of eccDNA in patients with MI, we identified a total of 8,283 eccDNAs using Circle-Seq in plasma samples from 5 patients with MI and 5 healthy controls (Fig. 1A). The Venn diagram illustrates that 3,993 eccDNAs were exclusive to patients with MI, 2,472 were unique to healthy controls, and 1,818 were common to both groups (Fig. 1B). Expression profiling revealed 1,424 eccDNAs with significant dysregulation in patients with MI compared to healthy controls, of which 1,109 were up-regulated and 315 were down-regulated (Fig. 1C). Interestingly, the genes associated with these up-regulated eccDNAs were significantly enriched to Gene Ontology (GO) terms related to “cell–cell adhesion mediated by cadherin (GO:0044331)” and “cell junction assembly (GO:0034329)” (Fig. 1D), whose roles in cardiovascular diseases have been intensively investigated. Moreover, Kyoto Encyclopedia of Genes and Genomes (KEGG) pathway analysis revealed that the differentially expressed eccDNAs were mainly involved in tight junction, leukocyte transendothelial migration, axon guidance, and circadian entrainment (Fig. 1E). The study further characterized and analyzed the expression frequency, length distribution, GC content, and genomic distribution of the eccDNAs, noting that they originated from various chromosomes, particularly chromosomes 1 and 2 (Fig. 1F). Size distribution analysis indicated that eccDNAs smaller than 500 bp were predominant in the plasma of both patients with MI and healthy individuals, with 2 prominent peaks at approximately 200 and 350 bp (Fig. 1G). Furthermore, our analysis revealed disproportionately elevated levels of CpG islands and comparatively reduced levels in the *Alu* region within the mapping results, suggesting a nonrandom formation of circular DNAs (Fig. 1H). In addition, the GC content of eccDNAs was observed to be higher than that of the immediate upstream or downstream flanking regions in both groups studied (Fig. 1I). These findings not only enhance our comprehension of the role of circular DNAs in MI but also underscore the potential significance of these structures in the context of infarction.

### Identification of MIRECD in MI

**Fig. 2.**
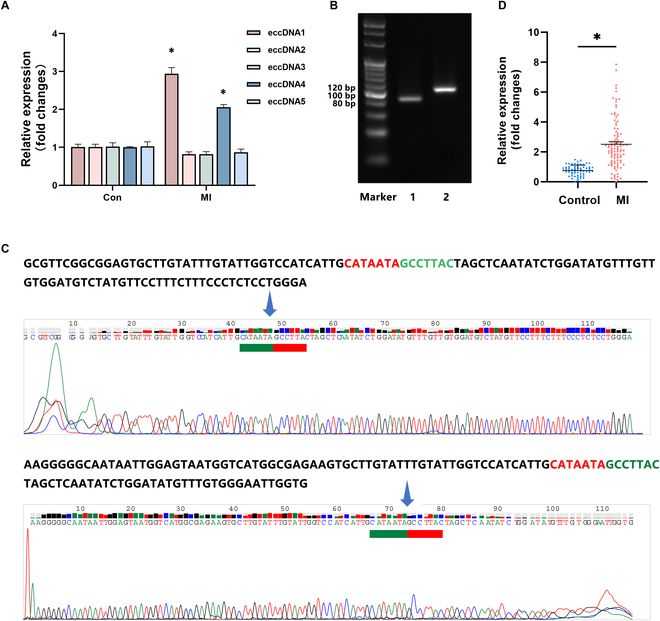
Selection and validation of eccDNAs in MI plasma. (A) qPCR results of 5 eccDNAs detected by high-throughput sequencing (eccDNA1: chr6:4774194–4774429, eccDNA2: chr1:217633935–217634137, eccDNA3: chr2:105476963–105477164, eccDNA4: chr16:66381718–66381911, and eccDNA5: chr14:22757334–22757523). (B) PCR results of 5 differentially expressed eccDNAs according to Circle-Map (1, eccDNA4; 2, eccDNA1). (C) Sanger sequencing validated the junctional site of MIRECD. (D) qPCR validation of MIRECD in patients with acute MI (*n* = 60) and healthy controls (*n* = 100). The data were expressed as means ± SEM. Differences between groups were analyzed using an unpaired *t* test, and *P < 0.05 was considered statistically significant. Con, the control group.

By using Circle-Map [18] with recommended filters (circle score ≥ 50, coverage at the start ≥ 0.33, coverage at the end ≥ 0.33, and no overlap with repeat elements), we identified 472, 447, 102, 440, and 895 circular DNAs in the 5 MI samples, respectively. Among the identified circular DNA genes, 239 were observed in at least 2 samples. To further reduce the list of circular DNA genes for experimental validation, we selected 26 genes that were identified in at least 3 samples from the MI group but were not found in any samples from the control group (Table S2). Among these genes, we finally chose 5 eccDNAs that were annotated to genes closely related to heart development and functions for downstream validation (chr1:217633935–217634137, chr2:105476963–105477164, chr6: 4774194–4774429, chr14:22757334–22757523, and chr16:66381718–66381911). Quantitative polymerase chain reaction (qPCR) confirmed that chr6:4774194–4774429 and chr16:66381718–66381911 were significantly up-regulated in MI (Fig. 2A). These 2 eccDNAs were then selected to confirm the presence of a cyclization site using Sanger sequencing (Fig. 2B). We successfully validated that chr6:4774194–4774429, named MIRECD, had a splicing site (CATAATAGCCTTAC), thus demonstrating its looping structure (Fig. 2C). The complete sequence of MIRECD is presented in Table 1. We further confirmed that MIRECD was highly expressed in the plasma of patients with MI using qPCR (Fig. 2D).

**Table 1.** MIRECD sequence

Name	Gene location	Sequence 5′–3’
MIRECD	chr6:4774194–4774429	GCCTTACTAGCTCAATATCTGGATATGTTTGTTGTGGATGTCTATGTTCCTTTCTTTCCCTCTCCTGGGCAACAATGGCAGTTTTGGAGACTCCTTGGAGACCACAGTACCATTACGCACATGGTGGAGCCCCTTGTGAATGCCTAAGCTTGTGGATGTTCTGAAGGGGGCAATAATTGGAGTAATGGTCATGGCGAGAAGTGCTTGTATTTGTATTGGTCCATCATTGCATAATA

### Functional study of MIRECD

**Fig. 3.**
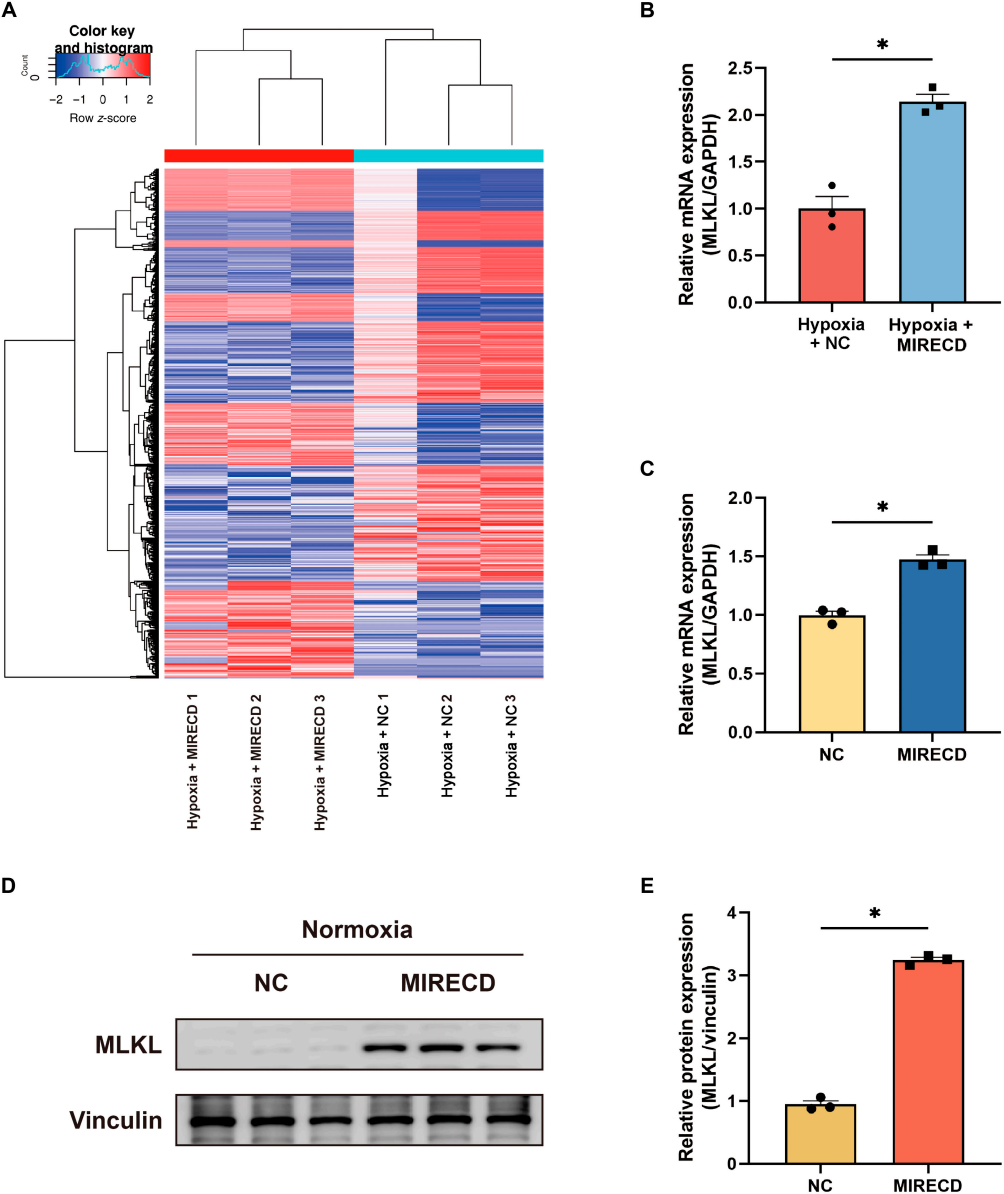
Overexpression of MIRECD up-regulated MLKL expression. (A) Heatmap of differential gene expression by RNA sequencing after MIRECD transfection. Relative MLKL mRNA levels after transfection with MIRECD under (B) hypoxic and (C) normoxic conditions. GAPDH, glyceraldehyde-3-phosphate dehydrogenase. (D) Expression of MLKL protein in transfected cells. (E) Bar graphs depict relative fold change of MLKL in AC16 cells. Each experiment was conducted in triplicate, and the results are presented as the means ± SEM. Group differences were assessed using an unpaired *t* test, *P < 0.05. *n* = 3.

AC16 cells were transfected with MIRECD and exposed to hypoxia before RNA sequencing for differential gene expression analysis. The findings are illustrated in a cluster heatmap (Fig. 3A). Mixed-lineage-kinase-domain-like pseudokinase (MLKL) expression significantly increased after MIRECD transfection, which was confirmed by reverse transcription (RT)-qPCR (Fig. 3B). To investigate the potential independence of this effect from hypoxia, we transfected AC16 cells with MIRECD and subsequently cultured them under normoxic conditions. The expression levels of MLKL mRNA and protein were assessed using RT-qPCR and Western blot, respectively (Fig. 3C to E).

**Fig. 4.**
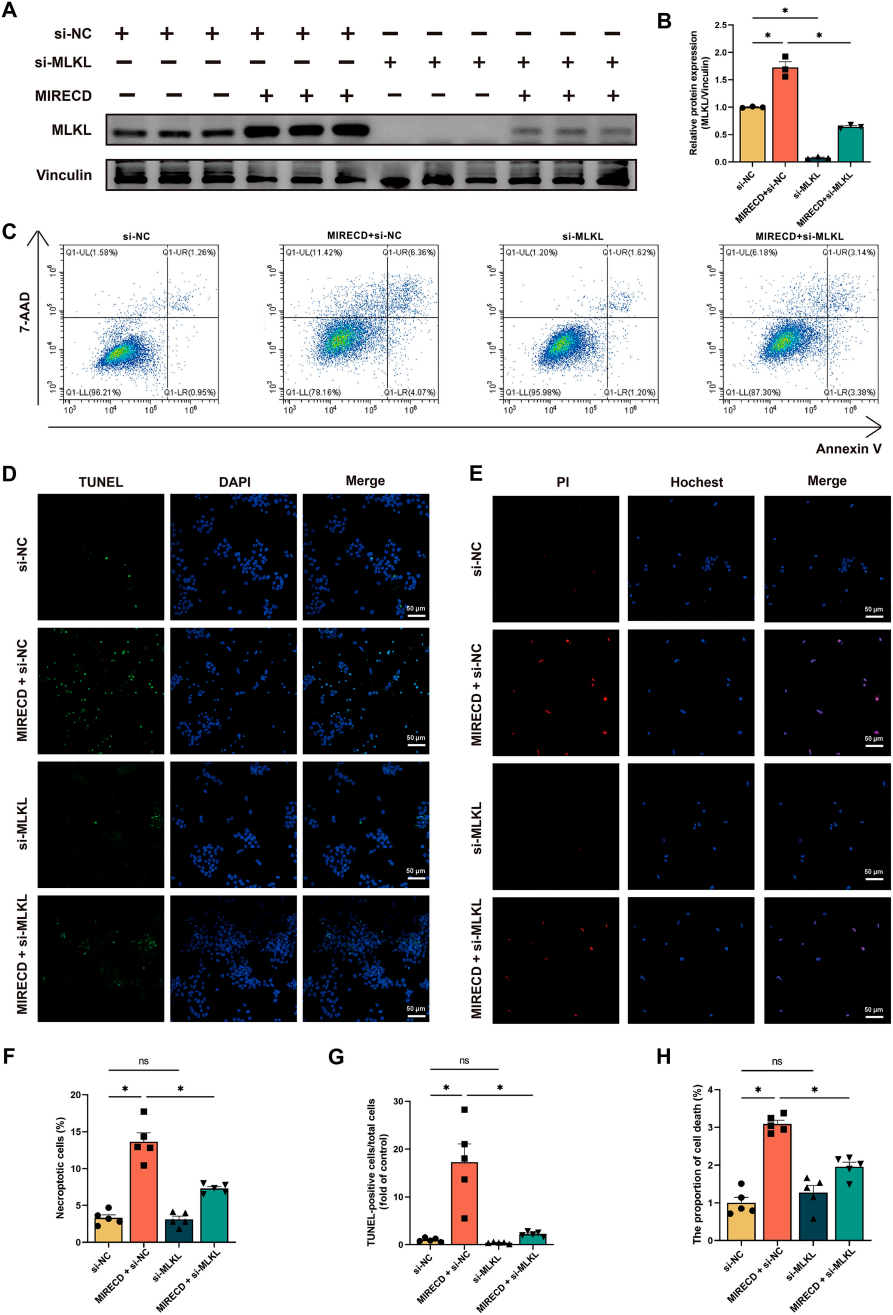
Overexpression of MIRECD could aggravate necroptosis via MLKL. (A) Western blot analysis of protein expression levels after siRNA transfection. *n* = 3. (B) Bar diagram shows MLKL protein expression level normalized to vinculin in each group. *n* = 3. (C) Necroptosis of AC16 cells after MIRECD transfection evaluated by flow cytometry. (D) Representative images of TUNEL assay results. Scale bars, 50 μm. (E) Representative images showing Hoechst–PI costaining in MIRECD-transfected cells. Scale bars, 50 μm. (F) Cells undergoing necroptosis were calculated and shown in histograms. (G) Bar graphs showing percentage of TUNEL-positive cells in different groups. (H) Bar graphs showing percentages of necroptotic cells (PI-positive, Hoechst-positive). Each experiment was conducted in triplicate, and the results are presented as the means ± SEM. Group differences were assessed using analysis of variance (ANOVA), followed by Tukey’s post hoc multiple comparison tests. *P < 0.05. ns indicated no significant difference. *n* = 5.

Furthermore, we investigated the role of MLKL in AC16 cells following knockdown of MLKL protein by small interfering RNA (siRNA) (Fig. 4A and B). Necroptosis was induced by MIRECD and partly inhibited by knockdown of MLKL, as shown by flow cytometry (Fig. 4C and F). Given the diverse morphological manifestations of necroptosis, we used alternative methods to assess this effect. Subsequent evaluation of necroptosis rates by terminal-deoxynucleotidyl-transferase-mediated deoxyuridine triphosphate nick-end labeling (TUNEL) assay and Hoechst–propidium iodide (PI) staining revealed that MIRECD transfection increased necroptotic cells (Fig. 4D, E, G, and H), and this effect was reversed by transfection with MLKL siRNA (si-MLKL). These findings indicated that MIRECD expression may influence programmed cell death through modulation of the necroptotic pathway, thus suggesting a potential mechanism by which eccDNA may exacerbate cardiomyocyte injury during MI.

### Cohort study of patients with MI

Given the high expression of MIRECD in patients with MI, we hypothesized that it could serve as a promising biomarker for the progression and prognosis of MI. We thus conducted a prospective study in 515 individuals to examine the predictive significance of MIRECD in MI. The baseline characteristics of the participants are shown in Table 2. Patients with MI were categorized into 2 groups based on the median MIRECD levels. Individuals with higher MIRECD levels were more likely to have previous occurrences of acute MI and HF. MIRECD was positively correlated with ST-elevation MI, Killip class, fasting blood glucose (FBG), cTnI, and BNP and inversely related with left ventricular ejection fraction (LVEF) and the use of statins and ezetimibe. The average duration of follow-up was 36 months, during which time 123 patients (23.88%) experienced adverse end-point events, including 32 cardiovascular deaths.

**Table 2.** Baseline characteristic of patients with AMI according to median levels of MIRECD

	All patients (*N* = 515)	MIRECD < 2.36	MIRECD ≥ 2.36	*P* value
Male	396 (76.9%)	193 (74.8%)	203 (79.0%)	0.260
Age, y	65.00 (55.00–73.00)	65.00 (56.00–72.00)	64.50 (55.00–74.00)	0.413
BMI	24.00 (23.00–27.00)	25.00 (23.00–27.00)	24.00 (23.00–27.00)	0.620
Previous history				
AMI	45 (8.7%)	12 (4.7%)	33 (12.8%)	<0.001
PCI	70 (13.6%)	33 (12.8%)	37 (14.4%)	0.607
HF	41 (8.0%)	5 (1.9%)	36 (14.0%)	<0.001
Hypertension	358 (69.5%)	179 (69.4%)	179(69.6%)	0.947
Diabetes	155 (30.1%)	71 (27.5%)	84 (32.7%)	0.201
Hyperlipidemia	46 (8.9%)	29 (11.2%)	17 (6.6%)	0.066
Smoking	278 (54.0%)	138 (53.5%)	140 (54.5%)	0.822
STEMI	222 (43.1%)	89 (34.5%)	133 (51.8%)	<0.001
Killip class > 1	168 (32.6%)	31 (12.0%)	137 (53.3%)	<0.001
LVEF	55.00 (46.00–63.00)	60.00 (55.00–65.00)	47.00 (41.00–56.00)	<0.001
Medical treatment				
Aspirin	432 (83.9%)	220 (85.3%)	212 (82.5%)	0.391
P2Y12 inhibitor	466 (90.5%)	230 (89.1%)	236 (91.8%)	0.300
Statins	502 (97.5%)	257 (99.6%)	245 (95.3%)	0.002
Ezetimibe	214.0 (42.5%)	119 (47.0%)	95 (37.8%)	0.037
B-blocker	363 (71.9%)	189 (73.8%)	174 (69.9%)	0.324
ACEI/ARB/ARNI	245 (47.6%)	125 (48.4%)	120 (46.7%)	0.690
Trimetazidine	212 (42.5%)	112 (44.3%)	100 (40.7%)	0.414
Laboratory tests				
Hb	134.00 (122.00–147.00)	134.00 (121.75–147.00)	134.00 (122.00–146.00)	0.469
TG	1.41 (1.02–2.02)	1.46 (1.06–2.10)	1.39 (1.02–1.98)	0.745
TC	4.12 (3.36–4.92)	4. 04 (3.31–4.89)	4.13 (3.41–4.91)	0.617
LDL	2.50 (1.85–3.26)	2.41 (1.73–3.22)	2.59 (1.96–3.28)	0.289
HDL	0.99 (0.84–1.18)	0.98 (0.82–1.17)	0.99 (0.85–1.20)	0.253
FBG	6.04 (5.20–7.35)	5.94 (5.16–7.14)	6.11 (5.21–7.62)	0.004
Scr	74.00 (61.00–87.00)	72.00 (60.00–86.25)	75.00 (61.00–87.00)	0.922
cTnI	3.73 (0.10–29.73)	16.78 (0.97–46.86)	39.85 (7.03–50.00)	<0.001
BNP	153.00 (48.00–312.00)	109.00 (28.75–245.75)	175.50 (66.00–372.00)	<0.001

Values are median (interquartile range) or *n* (%).

BMI, body mass index; ACEI, angiotensin-converting enzyme inhibitor; AMI, acute MI; STEMI, ST-elevation MI; ARB, angiotensin receptor blocker; ARNI, angiotensin receptor–neprilysin inhibitor; Hb, Hemoglobin; NT-proBNP, N-terminal pro-BNP; CRP, complement-reactive protein; TG, triglyceride; TC, total cholesterol; LDL, low-density lipoprotein; HDL, high-density lipoprotein; PCI, percutaneous coronary intervention; Scr, serum creatinine.

### Analysis of survival using the Kaplan–Meier method

**Fig. 5.**
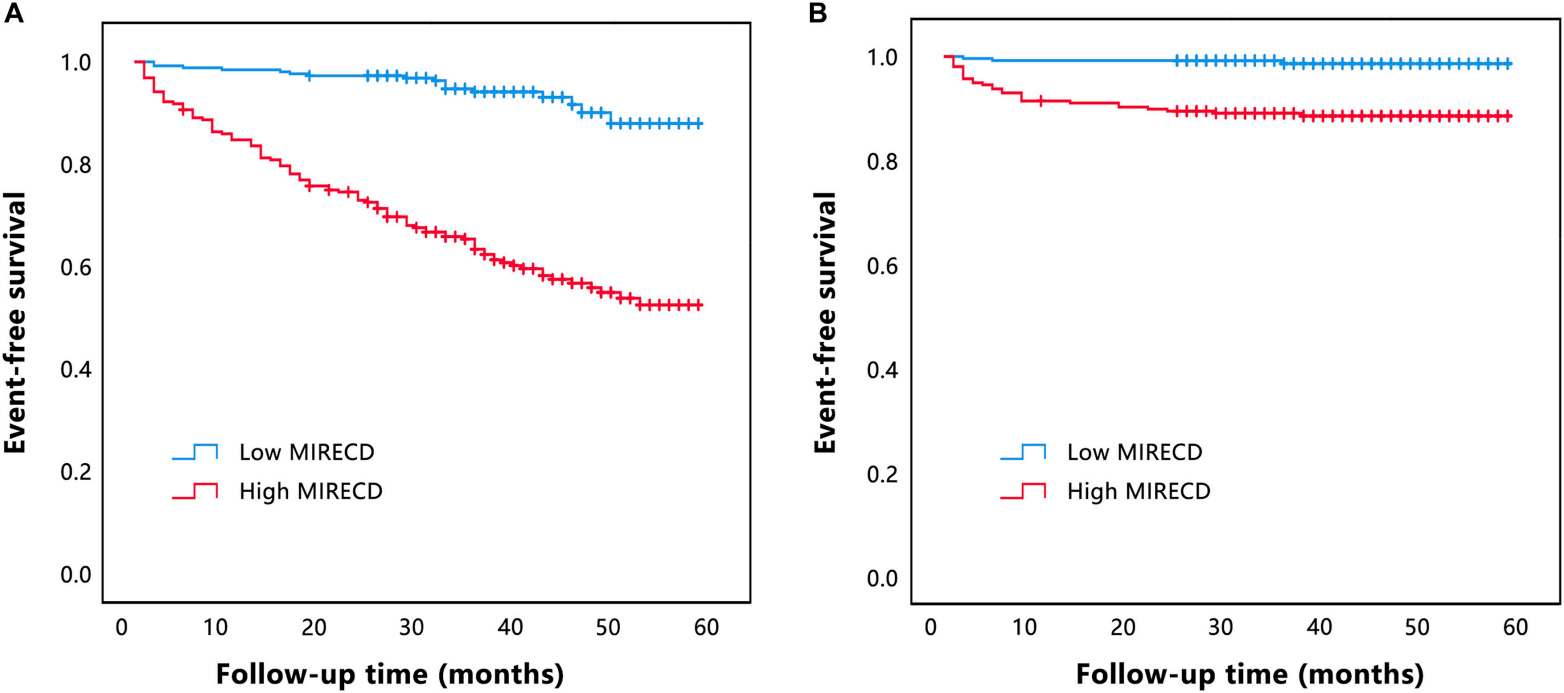
Kaplan–Meier survival curves. The event-free survival of MACEs (A) and cardiovascular mortality (B) in patients with acute MI, stratified according to median levels of plasma MIRECD.

We compared survival rates among patients with different plasma MIRECD expression levels using Kaplan–Meier analysis. Plasma MIRECD levels were a significant indicator of major adverse cardiac events (MACEs) and cardiovascular mortality in patients with MI (Fig. 5A and B). MIRECD levels above the median were associated with higher rates of MACEs (log-rank test, *P* < 0.001) and cardiovascular mortality (log-rank test, *P* < 0.001) compared with individuals with MIRECD levels below the median.

### Cox regression analysis

The Cox regression model for survival analysis indicated that age, previous histories of acute MI, HF, hypertension, and diabetes, Killip class, BNP, and MIRECD were individual predictors of MACE, which represents a combination of cardiovascular mortality, HF hospitalization, and recurrent acute MI. Multivariate Cox regression analysis also identified MIRECD as a significant predictor of MACE [hazard ratio (HR) = 2.72; 95% confidence interval (CI) = 2.02 to 3.67, *P* < 0.001), together with age, previous histories of acute MI, HF, and diabetes, and Killip class (Table 3). Harrell’s C-index for MACE increased from 0.862 (95% CI = 0.833 to 0.891) to 0.877 (95% CI = 0.851 to 0.904) (*P* < 0.001) after the addition of MIRECD to the reference model. Moreover, the model including MIRECD had better predictive value than that without MIRECD according to integrated discrimination improvement (IDI) and net reclassification improvement (NRI) (IDI = 0.0629, 95% CI = 0.0395 to 0.0864; categorical NRI = 0.2438, 95% CI = 0.1424 to 0.3452; continuous NRI = 0.8914, 95% CI = 0.7066 to 1.0761; all *P* < 0.001).

**Table 3.** Cox regression analysis for MACE in patients with AMI

	Univariable analysis	*P* value	Multivariable analysis	*P* value
	HR (95% CI)		HR (95% CI)	
Male	0.71 (0.48–1.06)	0.098	0.85 (0.49–1.46)	0.548
Age, y	1.04 (1.02–1.05)	<0.001	1.04 (1.02–1.06)	<0.001
BMI	0.97 (0.93–1.02)	0.270	0.98 (0.93–1.03)	0.435
Previous history				
AMI	6.65 (4.45–9.94)	<0.001	2.25 (1.36–3.71)	0.002
PCI	1.23 (0.75–2.00)	0.412	0.87 (0.52–1.46)	0.605
HF	8.35 (5.14–16.09)	<0.001	3.83 (2.29–6.38)	<0.001
Hypertension	1.76 (1.14–2.69)	0.010	1.00 (0.62–1.61)	0.983
Diabetes	2.88 (2.02–4.10)	<0.001	2.11 (1.40–3.16)	<0.001
Hyperlipidemia	0.78 (0.39–1.53)	0.468	1.66 (0.80–3.45)	0.178
Smoking	0.81 (0.57–1.16)	0.249	0.88 (0.54–1.43)	0.609
STEMI	1.25 (0.87–1.78)	0.226	1.16 (0.75–1.82)	0.504
Killip class > 1	5.24 (3.63–7.58)	<0.001	4.35 (2.82–6.73)	<0.001
Medical treatment				
Aspirin	0.80 (0.50–1.26)	0.333	1.58 (0.88–2.82)	0.124
P2Y12 inhibitor	0.86 (0.46–1.60)	0.623	0.66 (0.28–1.58)	0.349
Statins	0.47 (0.21–1.07)	0.074	0.80 (0.24–2.65)	0.719
B-blocker	0.96 (0.65–1.42)	0.836	1.24 (0.80–1.92)	0.341
ACEI/ARB/ARNI	0.95 (0.66–1.35)	0.756	1.13 (0.75–1.69)	0.558
LogcTnI	1.08 (0.92–1.27)	0.340	1.02 (0.81–1.28)	0.886
LogBNP	2.11 (1.51–2.95)	<0.001	1.13 (0.75–1.69)	0.569
MIRECD	3.77 (2.97–4.78)	<0.001	2.72 (2.02–3.67)	<0.001

## Discussion

**Fig. 6.**
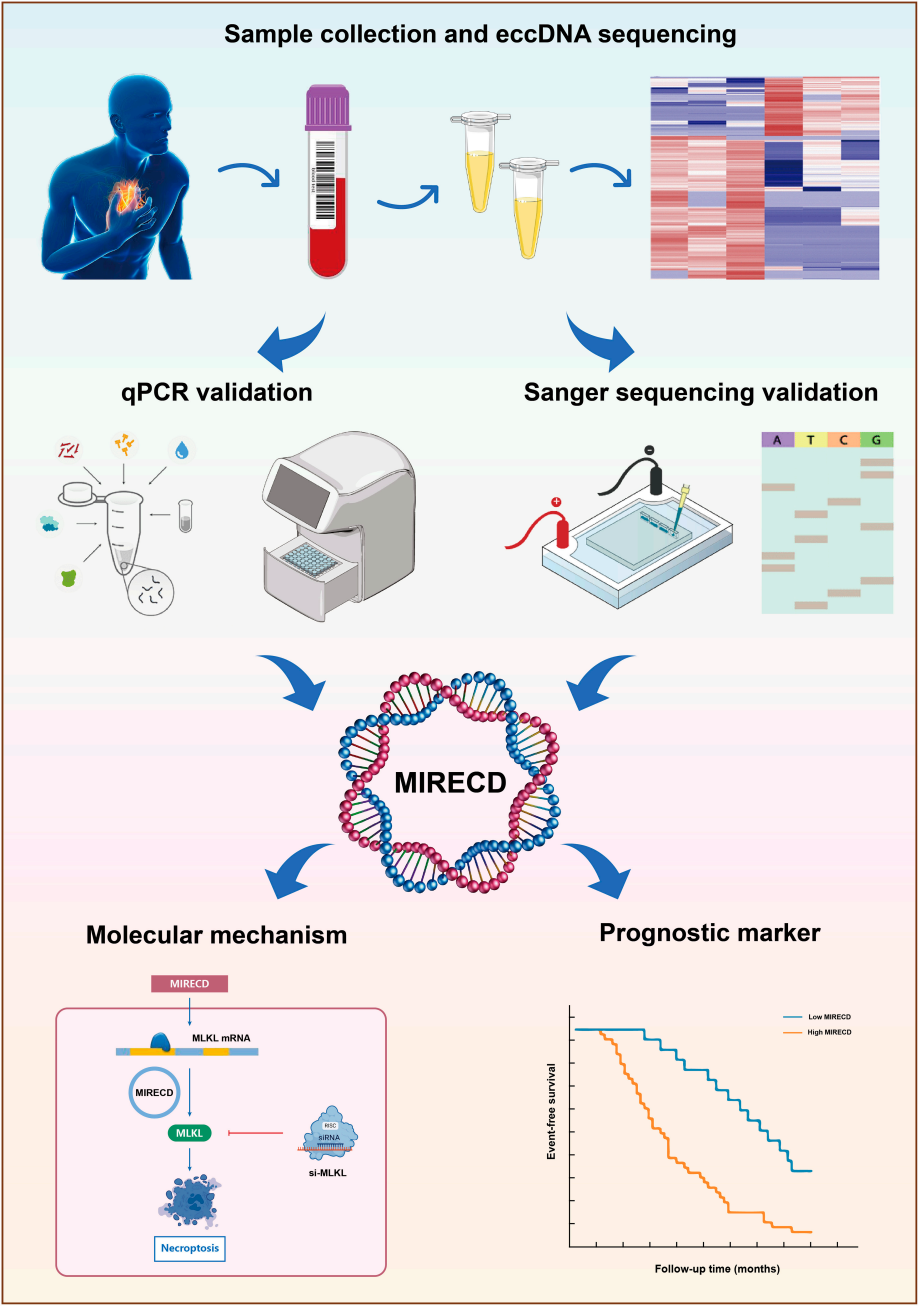
Schematic illustration of plasma eccDNA identification and analysis of downstream mechanisms. First, eccDNA was extracted from plasma and subjected to high-throughput sequencing for screening differentially expressed eccDNAs. Second, qPCR validation of identified eccDNAs in MI patients. Third, Sanger sequencing was performed to validate their looping structure. Fourth, the exploration of downstream molecular mechanism and the evaluation of the prognostic value of MIRECD.

We showed that MIRECD, a type of eccDNA, was highly expressed in patients suffering from MI, using high-throughput sequencing technologies. The sequencing results were further corroborated using qPCR. Considering its closed-loop structure, we detected the precise arrangement of bases by Sanger sequencing to confirm the specific cyclization site in MIRECD. Further cellular experiments revealed that MIRECD may regulate necroptosis by affecting MLKL gene expression in AC16 cells. In the prognosis analysis, patients with higher expression of MIRECD had a significantly increased incidence of MACEs, suggesting that MIRECD may serve as a biomarker to facilitate more accurate prediction of prognosis in patients with MI (Fig. 6).

Increasing attention is being paid to the function of eccDNAs, especially their application as biomarkers in liquid biopsy [19]. In 2017, Kumar et al. [20] proposed that plasma eccDNA was released from normal tissues and tumors into the circulation. The length of plasma eccDNA was longer before surgery in patients with lung and ovarian cancer. Sin et al. [21] demonstrated the existence of eccDNA in maternal plasma and found that the methylation levels of maternal eccDNAs were higher than those of fetal eccDNAs, which could be rapidly cleared from the maternal blood after delivery [22]. Moreover, numerous eccDNAs were recently identified in serum of patients with lung adenocarcinoma, and Xu et al. [23] showed that increased expression of CircD-PDZRN3 and decreased expression of CircD-LGR6 clearly distinguished between patients with lung adenocarcinoma and healthy controls. Notably, however, there have been no relevant reports on eccDNA in cardiovascular diseases. The current study accordingly identified a specific eccDNA, MIRECD in the plasma of patients with MI, and confirmed that it could serve as a prognostic biomarker of MI.

Our results suggested that the detrimental impact of MIRECD may be mediated via the activation of necroptotic pathways, potentially exacerbating cardiac dysfunction following MI. Previous studies, however, have tended to examine the overall joint effects of eccDNAs. Wang et al. [24] demonstrated that the circularity, instead of the sequence, triggered innate immune responses using eccDNAs synthesized in vitro. In a functional study, Wang et al. [25] isolated eccDNAs from proteasome-inhibitor-resistant cell lines and transfected them into proteasome-inhibitor-sensitive cell lines. In the current study, we investigated the specific functions of a particular eccDNA.

The concept of necroptosis, introduced in 2005, has garnered increasing attention, as a form of programmed cell death distinct from apoptosis [26]. Necroptosis exhibits similarities to necrosis in terms of its morphological and pathological characteristics but includes specific steps and signaling cascades [27]. As an executioner of necroptosis, MLKL protein can be activated by phosphorylation and then translocated to the plasma membrane, where its accumulation on the membrane disrupts the integrity of the phospholipid bilayer, ultimately leading to cell death [28]. The expression of MLKL protein is currently considered as a decisive factor affecting cell survival in necroptosis [29]. In a previous study by Zhou et al. [30], overexpression of MLKL in cells subjected to oxygen and glucose deprivation/reoxygenation significantly reduced cell proliferation through necroptosis. Coincidentally, our experiments revealed that MIRECD triggered necroptosis by up-regulating MLKL gene expression. These findings indirectly confirmed the hypothesis that MIRECD was produced during MI and might significantly affect MI progression via MLKL protein expression. Overall, the current study provides the first evidence of an interaction between an eccDNA and the necroptotic pathway; however, further studies are needed to determine how MIRECD regulates cardiomyocyte necroptosis during MI.

This study had some limitations. First, although we identified a new eccDNA in blood samples of patients with MI, there is currently no evidence for the existence of MIRECD in mice, which, thus, limits further investigations of the molecular mechanisms of MIRECD in MI through animal experiments. Second, although we demonstrated a potential interaction between MIRECD and the necroptotic pathway, further experimental validation of a direct interaction is still needed. Third, the interactions of MIRECD with other members of the necroptotic pathway and its ability to initiate other pathways that influence cardiomyocyte survival remain unknown. These results imply that other mechanisms may drive MIRECD-induced necroptosis, and further studies are needed to explore these underlying mechanisms.

In conclusion, our study identified a novel eccDNA, MIRECD, which may influence the process of MI via the necroptotic pathway. High levels of MIRECD were linked to MACEs in patients with MI, indicating its potential as a predictor for MI prognosis.

## Materials and Methods

### Patient enrollment and eccDNA sequencing

Patients with acute MI and healthy controls were recruited from the Second Affiliated Hospital of Nanjing Medical University and the Second Affiliated Hospital of Soochow University. Age- and sex-matched individuals were used as controls. Acute MI was diagnosed according to 2017 European Society of Cardiology Guidelines for the management of acute MI [31]. All participants gave written informed consent before joining the study.

Gene sequencing was performed using peripheral blood samples. All samples were centrifuged at 3,000*g* for 10 min at 4 °C and then stored at −80 °C. Plasma DNA was extracted using the QIAamp Circulating Nucleic Acid Kit. To eliminate linear DNA and enhance the concentration of eccDNA, the plasma DNA underwent digestion with Plasmid-Safe adenosine 5′-triphosphate (ATP)-dependent deoxyribonuclease (DNase) (Epicenter), followed by column purification with the MinElute Reaction Cleanup Kit (QIAGEN). To establish a DNA library, the eccDNAs were processed using the Nextera XT DNA Library Preparation Kit. The DNA library underwent sequencing on an Illumina NovaSeq 6000 system in 150-bp paired-end mode.

### Circle-Seq data analysis

Contaminated adapters and low-quality sequence were trimmed from the raw Circle-Seq files by cutadapt software [32]. The cleaned sequencing reads were aligned to the hg19 human genome reference by using bwa software [33]. We used the Circle-Map [18] to detect eccDNAs across the samples. The quantification of soft-clipped reads intersecting with breakpoints was performed using Samtools software (v 0.2) [34], serving as the basis for raw counts. For normalization purposes, edgeR software (v0.6.9) [35] was applied, facilitating the computation of fold changes and corresponding *P* values to discern differentially expressed eccDNA between the groups. Cleaned circular DNA output was then annotated using the Bedtools software (v2.27.1) [36]. The online tool Enrichr was next used for the GO analysis and KEGG analysis on the annotated genes.

### Sanger sequencing validation

eccDNAs chosen on the basis of the Circle-Seq data were subjected to experimental validation. DNA was extracted from the plasma, and linear DNA was removed as described above. To enhance the productivity, PCR amplification was conducted utilizing 2× PCR Master Mix (GenSeq Biotech Inc.). The samples were temporarily stored at 4 °C or used immediately for subsequent experiments. The PCR samples were loaded on 2% agarose gels and observed using an ultraviolet luminescent image analyzer. Purified PCR products were retrieved using a MiniBEST Agarose Gel DNA Extraction Kit Ver.4.0 (TaKaRa), according to the manufacturer’s guidelines. Sanger sequencing was conducted by CloudSeq Biotech Inc., and the obtained data were subsequently analyzed using Chromas software (version 2.6.4; Technelysium).

### Synthetic MIRECD preparation

MIRECD was prepared according to the Ligase Assisted Minicircle Accumulation procedure [24,37]. The MIRECD sequence was obtained from National Center for Biotechnology Information GenBank according to the sequence analysis results. All DNA sequences and primers were synthesized by Sangon Biotech. The sequences are listed in Table S3. Linear products with phosphorylation at 5′ were prepared with 2× Phanta Max Master Mix (Vazyme Biotech). Approximately 0.4 μg of each MIRECD template was included in 100 μl of reaction mixture containing Taq DNA ligase (New England Biolabs) and subjected to thermal cycling as follows: initial denaturation at 95 °C for 3 min, followed by annealing at 60 °C for 10 min, and nick ligation at 37 °C for 5 min, repeated for a minimum of 10 cycles. The circularized items were purified using the TIANquick Mini Purification Kit (TIANGEN) and subsequently digested with Plasmid Safe ATP-dependent DNase (Epicenter). The digested products were again recovered using the TIANquick Mini Purification Kit.

### Cell culture and transfection

The human cardiomyocyte cell line, AC16, was acquired from FuHeng Cell Center (FuHeng Biology, Shanghai, China) and cultured as described previously [38]. Briefly, AC16 cells were cultivated in Dulbecco’s modified Eagle’s medium (Gibco) containing 10% fetal bovine serum (Vivacell) and 1% penicillin/streptomycin solution (Beyotime) [39]. Cells in the hypoxia group were incubated in a hypoxia chamber (Thermo Fisher Scientific) under conditions of 1% O_2_, 5% CO_2_, and an N_2_-balanced atmosphere. AC16 cells were cultured under standard conditions with full medium until 70% to 80% confluence, after which they were transfected with MIRECD using Lipofectamine 3000 Reagent (Invitrogen). The cells were subsequently subjected to either hypoxic or normoxic conditions for 24 hours. For siRNA treatment, AC16 cells were transfected with si-MLKL or negative control siRNA (si-NC), which were obtained from GenePharma (Shanghai, China), using Lipofectamine 3000 24 hours before MIRECD transfection. The sequences are summarized in Table S4.

### RNA sequencing

RNA sequencing was conducted by CloudSeq Biotech (Shanghai, China). Total RNA underwent ribosomal RNA removal, followed by library construction with the GenSeq Low Input RNA Library Prep Kit. Quality and quantity checks were done using the BioAnalyzer 2100. An Illumina NovaSeq was used for sequencing, using 150-bp paired-end reads. Reads were matched to the reference genome through the use of hisat2 software (v2.0.4). HTSeq software (v0.9.1) [40] provided raw counts, and edgeR normalized data to identify differentially expressed mRNAs.

### RT-qPCR analysis

RT-qPCR was used to validate MIRECD expression using outward-facing primers in the sequencing samples. In addition, the presence of MIRECD was validated in a new cohort, including 100 patients with MI and 60 healthy individuals. Briefly, the pET-23a plasmid was added to plasma samples at a ratio of 1.2 × 10^6^ copies/ml and served as an internal control [41]. To measure gene expression, total RNA was extracted from AC16 cells, treated as above, using TRIzol reagent. The extracted total RNA was reverse-transcribed into cDNA using the HiScript III RT SuperMix for qPCR (+genomic DNA wiper) (Vazyme Biotech). The mRNA expression was detected using the QuantStudio 5 Real-Time PCR System (Thermo Fisher Scientific) in a reaction volume of 10 μl, including 2 μl of diluted template DNA, 5 μl of 2× qPCR SYBR Green Master Mix (CloudSeq), and 0.5 μl of each primer (10 μM). The reaction conditions were set according to the kit instructions. The fluorescent signal was monitored in the extension step of each cycle. Relative expression of eccDNA was determined by the 2^−ΔΔCt^ method. The primer sequences are listed in Table S5.

### Cell death assay

The cell death ratio was determined using an annexin V–phycoerythrin apoptosis detection kit. After transfection, AC16 cells were harvested using 0.25% trypsin (Beyotime), rinsed twice with ice-cold phosphate-buffered saline (PBS), counted, resuspended in binding buffer and analyzed by flow cytometry. Apoptotic cells were subsequently examined using a CytoFLEX S flow cytometer after incubation with fluorescent dyes, including annexin V and 7-amino-actinomycin, for 15 min in the dark. The cell death rate was determined by quantifying the proportions of cells in the lower and upper right quadrants of the plots, comprising early apoptotic cells and late apoptotic or necroptotic cells. Programmed cell death was also visualized using TUNEL and Hoechst–PI staining techniques. The reagents and kits utilized in this study included the One-Step TUNEL Apoptosis Assay Kit, Apoptosis and Necrosis Assay Kit, 4% paraformaldehyde solution (Biosharp), and 4′,6-diamidino-2-phenylindole (DAPI) staining solution (Beyotime). All experimental procedures were conducted in accordance with previous methods [42,43]. AC16 cells were seeded to 20-mm glass-bottom microscope dishes (NEST Biotechnology, Wuxi, China) and cultured as described before. For TUNEL staining, cells were fixed in paraformaldehyde, permeabilized, and incubated with TUNEL detection solution, followed by DAPI staining for nuclear visualization. Hoechst–PI staining was done by incubating cells with a staining buffer containing PI and Hoechst dyes for 30 min at 4 °C. Cells were washed with PBS before observation.

### Western blot

Western blot was carried out as described previously [44]. The membrane was incubated with antibodies specific to MLKL (ABclonal) and vinculin (ProteinTech), followed by exposure to horseradish-peroxidase-conjugated secondary antibody (Lianke). Images were subsequently captured using an eBlot Touch Imager (e-BLOT), and the band density was quantified using ImageJ software, using vinculin as an internal control for normalization.

### Clinical research

A total of 515 patients with consecutive acute MI admitted to the Second Affiliated Hospital of Nanjing Medical University and the Second Affiliated Hospital of Soochow University were included. Patients with a confirmed cancer diagnosis or in the final stages of kidney disease were excluded from the study. Data on demographics, clinical characteristics, and biochemical information were collected from the patients’ medical files. Each patient received standard medical care. Informed consent forms were signed and collected from all participants. The primary end point was MACEs, including cardiovascular mortality, hospitalization for HF, or recurrent acute MI. HF hospitalization was characterized as readmission to hospital with HF as the primary reason. The end points were acquired by thorough examination of the hospital database and by personally contacting each patient.

### Statistical analysis

Statistical analysis was conducted utilizing SPSS 26.0 (IBM SPSS). Categorical variables are presented as number (percentage) and continuous variables as median and upper and lower quartiles. Baseline differences in patients with MI were compared using χ^2^ and *t* tests. The normality of the distribution was assessed using the Shapiro–Wilk test. Kaplan–Meier analysis was performed to compare survival rates between patients with high and low expression of MIRECD, using the log-rank test. Univariate and multivariate Cox proportional hazard analyses were used to assess the connections between initial variables and MACEs. The Cox regression models for the primary end point included sex, age, body mass index, previous history of percutaneous coronary intervention, acute MI, HF, hypertension, diabetes, hyperlipidemia, smoking, ST-elevation MI, Killip class > 1, medical treatment with aspirin, P2Y12 inhibitors, statins, β-blockers, angiotensin-converting enzyme inhibitors, angiotensin receptor blockers, and angiotensin receptor and neprilysin inhibitors, as well as FBG, cTnI, BNP, and MIRECD.

Two multivariable models were developed to determine whether MIRECD could improve the prognostic ability in patients with MI. The initial model was based on conventional cardiovascular risk factors, including sex, age, body mass index, previous history of acute MI, HF, hypertension, diabetes, hyperlipidemia, smoking, and Killip class > 1. In addition to these variables, the second model included the variable of MIRECD expression. We evaluated the enhanced discriminatory power of MIRECD for predicting MACEs using Harrell’s C-index. The overall reclassification improvement was evaluated by NRI and IDI using the R package PredictABEL. To avoid subjectivity, the choice of cutoff values for the categorical NRI was based on the average incidence, half the incidence, and twice the incidence. The continuous (category-free) NRI was also calculated as a reference [45,46]. Statistical analysis on functional study was performed using GraphPad Prism9 software (https://www.graphpad.com). A statistically significant result was determined if the 2-tailed *P* value was <0.05.

## Data Availability

The data that support the findings of this study are available from the corresponding author upon reasonable request.
