# Supplementary material for: Extrachromosomal Circular DNA MIRECD Enhances Necroptosis and Predicts Prognosis of Myocardial Infarction
Source: Research (Wash D C). 2025 Aug 8;8:0803. doi: 10.34133/research.0803 (PMC12332259; doi:10.34133/research.0803)
Supplement: Supplementary 1 — Tables S1 to S5 [file research.0803.f1.docx]

# Supplementary Materials

**Table S1.** Basic information of sequencing participants

|  | **MI** | **Conrol** | **P value** |
| --- | --- | --- | --- |
| **BMI (kg/m^2^)** | 25.68 ± 1.62 | 24.66 ± 1.50 | NS |
| **SBP (mmHg)** | 128.20 ± 12.58 | 131.00 ± 11.58 | NS |
| **DBP (mmHg)** | 66.20 ± 6.34 | 67.80 ± 8.32 | NS |
| **HR (bpm)** | 83.40 ± 11.48 | 72.60 ± 6.58 | NS |
| **Hb(g/L)** | 145.20 ± 14.27 | 138.00 ± 12.19 | NS |
| **NT-proBNP (pg/mL)** | 2871.20 ± 931.93 | 1104.80 ± 297.16 | 0.004 |
| **cTnT (pg/mL)** | 1481.00 ± 664.12 | 46.00 ± 28.24 | 0.001 |
| **Creatinine (μmol/L)** | 111.40 ± 18.70 | 106.80 ± 14.17 | NS |
| **CRP (mg/L)** | 19.28 ± 9.51 | 7.52 ± 3.31 | 0.031 |
| **TG (mmol/L)** | 2.81 ± 1.02 | 2.30 ± 0.70 | NS |
| **TC (mmol/L)** | 5.48 ± 0.92 | 4.33 ± 0.99 | NS |
| **LDL (mmol/L)** | 3.36 ± 0.83 | 2.73 ± 0.46 | NS |
| **HDL (mmol/L)** | 0.90 ± 0.23 | 0.79 ± 0.20 | NS |
| **FBG (mmol/L)** | 6.76 ± 1.24 | 6.66 ± 0.63 | NS |
| **HbA1c (%)** | 6.28 ± 0.86 | 6.06 ± 0.63 | NS |
| **Homocysteine (μmol/L)** | 13.52 ± 5.03 | 10.96 ± 3.19 | NS |

BMI = body mass index; SBP = systolic blood pressure; DBP = diastolic blood pressure; HR = heart rate; Hb = Hemoglobin; NT-proBNP = N-terminal pro b-type natriuretic peptide; cTNT = cardiac troponin T; CRP = C-reactive protein; TG = triglyceride; TC = total cholesterol; LDL = low density lipoprotein; HDL = high-density lipoprotein; FBG = fasting blood glucose; HbA1c = hemoglobin A1c.

**Table S2.** Genes have been identified in at least 3 samples

|  | **Gene name** | **Sample 1** | **Sample 2** | **Sample 3** | **Sample 4** | **Sample 5** | **Sum** |
| --- | --- | --- | --- | --- | --- | --- | --- |
| **chr7: 92314578-92314770** | **FAM133B** | 1 | 0 | 1 | 1 | 1 | 4 |
| **chr16: 9571193-9571395** | **LINC01195** | 1 | 0 | 1 | 1 | 1 | 4 |
| **chr7: 77751839-77752208** | **RPL13AP17** | 0 | 1 | 1 | 1 | 1 | 4 |
| **chr12: 99255125-99255314** | **ANKS1B** | 1 | 0 | 0 | 1 | 1 | 3 |
| **chr1: 207249928-207250028** | **C4BPB** | 0 | 0 | 1 | 1 | 1 | 3 |
| **chr16: 66381718-66381910** | **CDH5** | 1 | 0 | 0 | 1 | 1 | 3 |
| **chr6: 4774243-4774445** | **CDYL** | 0 | 1 | 0 | 1 | 1 | 3 |
| **chr12: 96982016-96982211** | **CFAP54** | 1 | 1 | 0 | 0 | 1 | 3 |
| **chr18: 77532438-77532501** | **CTDP1** | 0 | 0 | 1 | 1 | 1 | 3 |
| **chr14: 22757334-22757523** | **DAD1** | 0 | 0 | 1 | 1 | 1 | 3 |
| **chr2: 116032511-116032710**  **chr2: 1165358-1165587** | **DPP10** | 0 | 1 | 0 | 1 | 1 | 3 |
| **chr12: 63833417-63833616** | **DPY19L2** | 0 | 0 | 1 | 1 | 1 | 3 |
| **chr12: 110125957-110126156** | **FAM222A** | 1 | 1 | 0 | 0 | 1 | 3 |
| **chr1: 217633935-217634137** | **GPATCH2** | 0 | 0 | 1 | 1 | 1 | 3 |
| **chr10: 33384362-33384462** | **ITGB1** | 0 | 1 | 1 | 1 | 0 | 3 |
| **chr10: 91888447-91888540** | **LINC01375** | 0 | 0 | 1 | 1 | 1 | 3 |
| **chr20: 22623749-22623915** | **LINC01384** | 0 | 0 | 1 | 1 | 1 | 3 |
| **chr7: 157312495-157312599** | **LOC101927914** | 0 | 0 | 1 | 1 | 1 | 3 |
| **chr1: 30327985-30328175** | **LOC101929406** | 1 | 1 | 1 | 0 | 0 | 3 |
| **chr11: 41160639-41161015** | **LRRC4C** | 0 | 0 | 1 | 1 | 1 | 3 |
| **chr2: 206457666-206457856** | **NRP2** | 1 | 1 | 0 | 0 | 1 | 3 |
| **chr1: 89175217-89175530** | **PKN2-AS1** | 0 | 1 | 1 | 0 | 1 | 3 |
| **chr2: 105476963-105477164** | **POU3F3** | 0 | 1 | 0 | 1 | 1 | 3 |
| **chr12: 3427786-3427837** | **PRMT8** | 0 | 0 | 1 | 1 | 1 | 3 |
| **chr10: 90254106-90254309** | **RNLS** | 0 | 0 | 1 | 1 | 1 | 3 |
| **chr12: 15994879-15995423** | **STRAP** | 0 | 0 | 1 | 1 | 1 | 3 |

**Table S3.** Primer and template sequence for MIRECD synthesis

| **Template for MIRECD synthesis** | |
| --- | --- |
| **MIRECD-line1** | GCCTTACTAGCTCAATATCTGGATATGTTTGTTGTGGATGTCTATGTTCCTTTCTTTCCCTCTCCTGGGCAACAATGGCAGTTTTGGAGACTCCTTGGAGACCACAGTACCATTACGCACATGGTGGAGCCCCTTGTGAATGCCTAAGCTTGTGGATGTTCTGAAGGGGGCAATAATTGGAGTAATGGTCATGGCGAGAAGTGCTTGTATTTGTATTGGTCCATCATTGCATAATA |
| **MIRECD-line2** | CGCACATGGTGGAGCCCCTTGTGAATGCCTAAGCTTGTGGATGTTCTGAAGGGGGCAATAATTGGAGTAATGGTCATGGCGAGAAGTGCTTGTATTTGTATTGGTCCATCATTGCATAATAGCCTTACTAGCTCAATATCTGGATATGTTTGTTGTGGATGTCTATGTTCCTTTCTTTCCCTCTCCTGGGCAACAATGGCAGTTTTGGAGACTCCTTGGAGACCACAGTACCATTA |

| **PCR primer sets to amplify template** | |
| --- | --- |
| **MIRECD-line1-F** | /5Phosphorylation/-GCCTTACTAGCTCAATATCTGG |
| **MIRECD-line1-R** | /5Phosphorylation/-TATTATGCAATGATGGACCAATACAA |
| **MIRECD-line2-F** | /5Phosphorylation/-CGCACATGGTGGAGCCCC |
| **MIRECD-line2-R** | /5Phosphorylation/-TAATGGTACTGTGGTCTCCAAG |

**Table S4.** siRNA sequences used in this study

| **siRNA** | **positive-sense strand** | **negative-sense strand** |
| --- | --- | --- |
| si-MLKL | GAAGCAUAUUAUCACCCUUTT | AAGGGUGAUAAUAUGCUUCTT |

**Table S5.** Sequence of primers in this study

| **Genes** | **Orientation** | **Sequence 5′-3’** |
| --- | --- | --- |
| chr16:66381718-66381911 | 1-F | CTTATGCATGGGCAAGACCT |
|  | 1-R | TTCACTGCCTCCACTCTCCT |
| chr2:105476963-105477164 | 2-F | CTGGCAAGTCTGTGCGATAA |
|  | 2-R | GGATTAGCAAGGCTGATGGA |
| chr14:22757334-22757523 | 3-F | TGATCCCATCTACCAAATAGTCAA |
|  | 3-R | TGTGAGTTTGTTATCCTACCATGT |
| chr6:4774194-4774429 | 4-F | AAGGGGGCAATAATTGGAGT |
|  | 4-R | CCCAGGAGAGGGAAAGAAAG |
| chr1:217633935-217634137 | 5-F | TCACAATTATTGAAAATTCCCCTTA |
|  | 5-R | TGCTCCATATGTGCCAACTC |
| pET-23a+ | 6-F | GTCTTGAGTCCAACCCGGTA |
|  | 6-R | GCCTACATACCTCGCTCTGC |
| GAPDH | 7-F | CAGGAGGCATTGCTGATGAT |
|  | 7-R | GAAGGCTGGGGCTCATTT |
| MLKL | 8-F | TTTGGAATCGTCCTCTGGGAAATCG |
|  | 8-R | GCTCCTGCTGCCGCTTCAC |
| HSPA1A | 9-F | AAGAACGCCCTGGAGTCCTACG |
|  | 9-R | CCACGAGATGACCTCTTGACACTTG |
